# Closing the gap: National Argentinian discussion forum on paediatric brain tumours

**DOI:** 10.3389/fonc.2023.1185766

**Published:** 2023-05-18

**Authors:** Lorena V. Baroni, Nicolas Fernández Ponce, Candela Freytes, Francisco R. Maldonado, Natalia Pinto, Javier González Ramos, Fabiana Lubieniecki, Pedro Zubizarreta, Daniel Alderete

**Affiliations:** ^1^ Service of Haematology/Oncology, Hospital Juan P. Garrahan, Buenos Aires, Argentina; ^2^ Service of Diagnostic Imaging, Hospital Juan P. Garrahan, Buenos Aires, Argentina; ^3^ Service of Radiotherapy, Hospital Juan P. Garrahan, Buenos Aires, Argentina; ^4^ Service of Neurosurgery, Hospital Juan P. Garrahan, Buenos Aires, Argentina; ^5^ Service of Pathology, Hospital Juan P. Garrahan, Buenos Aires, Argentina

**Keywords:** brain tumour, LMIC (low- and middle-income countries), strategy, outcome, CNS - central nervous system

The burden of Central Nervous System (CNS) tumours is further compounded by the fact that they require highly specialised and skilled multidisciplinary care, including access to modern neuroimaging, neurosurgery, neuropathology and molecular biology, radiotherapy and chemotherapy, which may not be widely available in an integrated manner in large parts of low-middle income countries (1–3). Delay in diagnosis and initiation of treatment, the lack of systematic staging and standardised treatment all over the country and the limited access to high complexity medical centres could have a significant influence on outcomes in developing countries (4, 5). In Argentina the incidence of paediatric CNS tumours is approximately 260 new cases (26 cases per million children) per year. Sixty two percent of them needed to migrate to high complexity medical centres for treatment between 2012-2019 (6). About 50% of paediatric brain tumours were treated at “Prof. Dr. Juan P. Garrahan”; a premier reference centre for the care of high complexity child pathologies throughout Argentina. Here the healthcare organisation is based on progressive care with a hierarchy of interdisciplinary activity and an integrated approach supported by the highest technological development (such as 1·5 T Magnetic Resonance Imaging scanners and medical linear accelerators for radiotherapy treatment) and scientific-technical level of its human resources. Although overall survival of brain tumours has improved over the years in this country, it is still lower than in high income countries. Furthermore, considerable mortality reduction was achieved in Hospital Garrahan during the last 10 years, being 61·8 percent of mortality in 2000 vs. 32·4 in 2018. However, this survival improvement was not followed by the rest of the country bringing to light that new strategies were urgently needed.

Since December 2020, a highly qualified multidisciplinary team (including neuro-oncologists, pathologists, radiologists, radiotherapists and neurosurgeons) from Hospital Garrahan has implemented a network and a remote communication strategy for early referral, accurate diagnosis and staging, aiming to provide adequate treatment of children with brain tumours between Hospital Garrahan and other centres in Argentina. Biweekly (twice per month) interactive multidisciplinary meetings were established between private/public medical centres across the country and Hospital Garrahan. This was set up as a prospective qualitative-quantitative study, with interventions and registration of times of compliance.

The active participation of private and public centres covering approximately 96·6% of the country’s population was achieved; being connected up to 40 working groups simultaneously. One hundred twenty five new CNS patients (67 during the first year; 58 during the second year) from 21 provinces (85% of the country) were discussed in a multidisciplinary manner during the first 24 months of this project; 7,2% regarding diagnosis advice, 52% for treatment definition and 40,8% for both. Fifty-three tumour samples required a pathology analysis/review at Hospital Garrahan; 43.39% for initial diagnosis, 50.94% for second review (changing the initial diagnosis in 29.6% cases) and 5.6% for molecular workup. Only 34 patients required a referral to a centre of high complexity; 45% for radiotherapy, 45% for surgical issues and 10% for high dose chemotherapy with stem cells rescue. More than 250 brain and spine MRI scans were reviewed to improve tumour staging and oncology management. After the multidisciplinary meetings, the final treatment plan differed in 47,5% from the initial proposal; 27,6% due to MRI reviewed results and 72,3% regarding oncological discussion. The most frequently implemented therapeutic changes after the discussions were the selection of chemotherapy strategies in 51.3%, followed by neurosurgical intervention in 32.8% and radiotherapy requirement and prescription (field and dose) in 15.7%. Twenty-five patients needed multiple discussions (range 2-4) regarding follow up or after changes in management to guarantee the continued benefit of this recommendation.

On the other hand, we found out the principle barriers for proper diagnosis and treatment in centres of our network. The lack of highly experienced subspecialised neuroradiologists and oncologists in brain tumours and the limited infrastructure, including neurosurgical, pathology and paediatric radiotherapy facilities are the main limitations.

The exponential benefit of this strategy was established, showing to be a useful and fundamental tool to be implemented as a national health policy. The success of the implementation was marked by acceptance, appropriation, feasibility, adherence, coverage and sustainability. Our experience showed that multidisciplinary team management with high level expertise is key in countries with limited resources. Although several years will be needed to see the real survival impact of this strategy on outcomes, it pretends to bridge the gap between high complexity centres and the local community, achieving diagnosis and treatment equity and access opportunity. Furthermore, a gradual gain of knowledge and experience in brain tumour management was shown over these 2 years, changing the main discussion topic from differential diagnosis and proper tumour staging during the first year to treatment strategies and local implementation in the second year.

Finally, we would propose this remote care modality as a state policy in the near future, going up to a higher scale and being expanded into a broader, more far-reaching practice/policy. Actually, a national oncological coordinator centre is being established to be implemented shortly. Importantly, this model may be applied not only to other areas of paediatric cancer care but also to any other area in which a knowledge and skill gap can be identified.

## Author contributions

Conceptualisation: LB, CF, NF, NP, FL, JG, FM, and DA; Methodology: LB and NF; Writing –Review and Editing: LB, PZ, and DA; Project Administration: LB and DA; Supervision: DA and PZ. All authors contributed to the article and approved the submitted version.
